# Correlation between Microstructure and Mechanical Properties of Heat-Treated Novel Powder Metallurgy Superalloy

**DOI:** 10.3390/ma15134524

**Published:** 2022-06-27

**Authors:** Xianjue Ye, Biaobiao Yang, Jiantao Liu, Yunping Li

**Affiliations:** 1 State Key Lab for Powder Metallurgy, Central South University, Changsha 410083, China; yexianjue@csu.edu.cn (X.Y.); biaobiaoyang@csu.edu.cn (B.Y.); 2 IMDEA Materials Institute, C/Eric Kandel 2, Getafe, 28906 Madrid, Spain; 3 Department of Materials Science, Universidad Politécnica de Madrid, Escuela Técnica Superior de Ingenieros de Caminos, 28040 Madrid, Spain; 4 High Temperature Material Research Institute, Central Iron and Steel Research Institute, Beijing 100081, China

**Keywords:** PM superalloy, solution treatment, high temperature, tensile, oxidation

## Abstract

In this work, the quantification of key microstructural features like γ′ size morphology distribution, grain size, and localized stress distribution, especially near a fracture, were coupled with mechanical properties under various temperatures in Ni-base powder metallurgy superalloys subjected to sub-solvus or super-solvus heat treatments. Compared to super-solvus heat-treated alloy, sub-solvus heat-treated superalloy with a finer grain size exhibited higher ductility/strength at 550 °C, whilst adverse trend was observed at higher temperatures (750 and 830 °C). Besides, for both alloys, the strength and ductility decreased with the decrease in strain rate, resulting from oxidation behavior. Larger grain size or less grain boundary density can facilitate the retardation of oxidation behavior and weaken the propensity of early failure at higher temperatures.

## 1. Introduction

Powder metallurgy (PM) Ni-base superalloys are widely used as turbine discs in aero engines, owing to their outstanding and comprehensive performance at high temperatures [1,2,3,4,5,6,7]. The development of PM superalloy can be divided into four generations, such as René95 [8] representing the first generation of PM superalloy, René88DT [9] for the second generation, and René104 [10] and RR1000 [11] representing the third generation of PM superalloy. For the requirements of the next generation aero engine, the next generation PM superalloy needs a higher level of comprehensive performance in areas such as high temperature/strength, high damage tolerance, etc. The addition of γ′ formers such as Al, Ti, Ta, and Nb needs to be increased to obtain enough γ′ fraction in the superalloy [12]. At the same time, TCP formers like Mo, W, etc. need to be reduced to maintain phase stability at high temperatures [2]. A novel PM superalloy was designed in the present study by considering the composition-property and calculated phase diagrams to obtain specific aspects of performance such as the strength, working temperature, density, oxidation resistance, and so on.

PM Ni-base superalloys are typically structured with a solid solution matrix (γ) and a secondary γ′ phase embedded in the matrix [2,13,14,15]. The γ′ phase precipitates could impede the movements of dislocations and they make the main contribution to the strength of superalloy [16,17]. Other microstructural factors such as grain size, carbides, topologically close pack (TCP) phases, etc., which are strongly correlated with the heat treatment process, were reported to have tremendous influence on the mechanical performance of PM Ni-base superalloy [18,19,20,21]. Therefore, tailoring the microstructure by heat treatment is an important way to obtain the target mechanic performance of superalloy.

For this, the correlation among heat treatment parameters, microstructural features, and the mechanical performance of Ni-base PM superalloy has drawn significant interest from researchers, particularly to meet the ever-growing high temperature performance requirement [16,17,22,23,24,25,26]. With the aid of high-throughput methodology, Wu et al. reported that a lower cooling rate can accelerate the growth of the γ′ phase [16,17]. Gai et al. carried out a solution treatment with a temperature range from 1100 °C to 1140 °C on PM superalloy and found the fraction of the primary γ′ decreased and the fraction of the secondary γ′ increased with the solution treatment temperature increasing [27]. Similar results have also been found by Wan et al., who conducted different solution treatment procedures on U780Li. Additionally, abnormal grain growth can be observed after heat treatment at 1150 °C [28]. Atabay et al. produced Rene 41 superalloy by laser powder bed fusion followed by sub-solvus and super-solvus treatments. The strength of the alloy after super-solvus was found to be higher than that after sub-solvus at 760 °C [29]. Vaunois et al. carried out both sub-solvus and super-solvus treatments on UDIMET 720Li superalloy and found that the ambient mechanical performance of the sub-solvus heat-treated superalloy was higher than that of the super-solvus one [24]. A similar phenomenon was also observed by Kozar et al. [30]; that is, the sub-solvus heat-treated IN100 superalloy exhibited superior mechanical performance to the super-solvus one at a low temperature (<600 °C). This was generally ascribed to the low mean free path of dislocations in fine grains at a low temperature, also known as the Hall–Petch relationship [30,31,32]. However, it must be mentioned that next generation PM superalloy have to function at a higher temperature (>850 °C), at which grain boundary sliding will dominate the deformation [31,33,34,35]. Additionally, fine-grained alloys have an occurrence of grain boundary sliding at high temperatures and are generally associated with decreased strength compared to alloys with large grains [36,37]. An obvious decline in yield strength of sub-solvus heat-treated IN100 can be observed when the testing temperature rises from 600 to 800 °C [30]. However, the correlation between the microstructure and the mechanical properties at high temperatures of the PM superalloy has not been studied systematically and the mechanism is not deeply understood.

In the present study, super-solvus and sub-solvus treatments were both carried out on a novel Ni-base PM superalloy. The mechanical testing was conducted at various temperatures and the deformation mechanism was analyzed in detail by SEM and EBSD techniques. This research is intended to pave the way for developing a next generation superalloy with enhanced comprehensive performance.

## 2. Materials and Methods

The nominal composition of powder metallurgy superalloy is tabulated in Table 1. The master alloy was prepared by vacuum horizontal continuous casting (VHCC) and the alloyed powder was manufactured by electrode induction melting gas atomization (EIGA). The powder was then consolidated through hot isostatic pressing (HIP) at 1180 °C/130 MPa for 4 h. The as-HIP ingot was subjected to hot extrusion at 1120 °C with an area reduction of 5:1. After that, isothermal forging at 1100 °C was carried out to produce the turbine disc. Then, isothermal forging was carried out. The γ’ solvus temperature was calculated to be 1142.29 °C by Thermal-Calc software using the TTNi8 data base. Two solution treatments were conducted to produce superalloy with different levels of grain size, including the super-solvus at 1180 °C/2 h + oil cooling and the sub-solvus at 1120 °C/2 h + oil cooling. The alloys after the aforementioned treatments were then subjected to aging as 870 °C/4 h + air cooling → 760 °C/16 h + air cooling. The specimens after super-solvus and sub-solvus treatment were hereafter referred to as SH and SL, respectively.

The high temperature mechanical performance was performed by using an electronic testing machine at 550, 750, and 830 °C. The testing samples were cut with a gauge length of 8 mm, a gauge width of 3.4 mm, and a gauge thickness of 3 mm by using an electric discharge machine (EDM). The strain rate was set to be 10^−3^ s^−1^, and mechanical testing at 750 °C under a lower strain rate (10^−4^ s^−1^) was also performed for both specimens.

A scanning electron microscope (SEM; FEI Quanta 650) was used to observe morphologies before and after mechanical testing. Specimens before testing were electrically etched in a solution of 45%H_2_SO_4_, 43% HNO_3_, and 12% H_3_PO_4_ with a voltage of 5 V for 20 s at room temperature for γ′ morphology observation. Electron backscatter diffraction (EBSD; Oxford Instrument plc, NordlysMax2) measurements were performed to obtain the microstructures perpendicular to the tensile direction near fracture after mechanical testing. Based on the respective grain size distribution, the step size of SH and SL for EBSD measurement was set to be 1 and 0.1 μm, respectively.

## 3. Results

**Figure 1:**
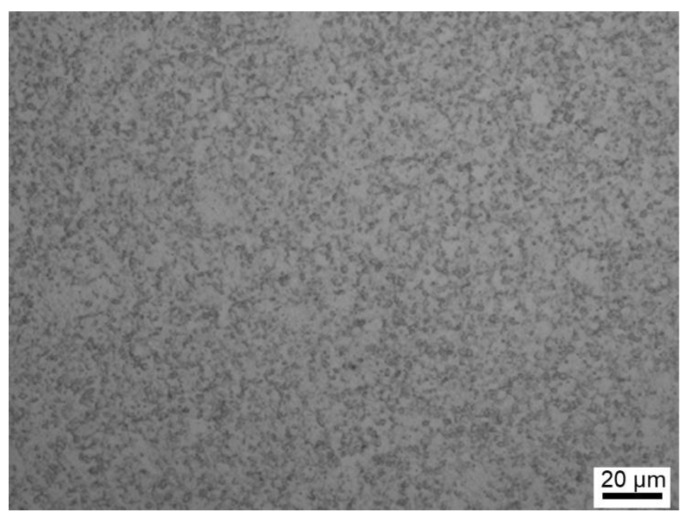
The microstructure of PM superalloy before heat treatment taken by optical microscopy.

**Figure 2:**
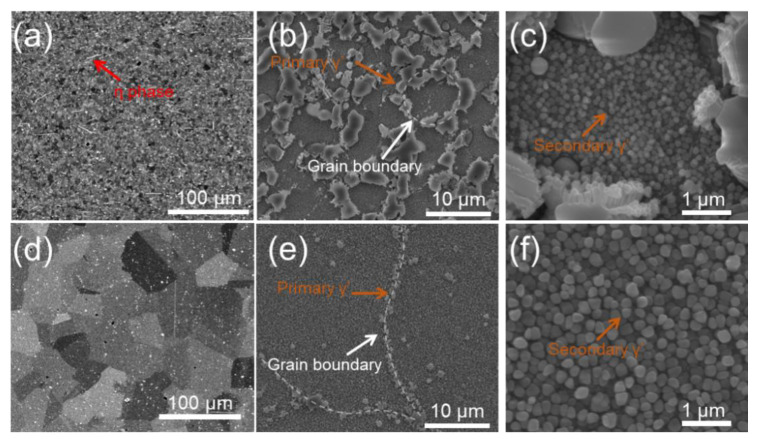
BSE images of microstructures (**a**,**d**) and SE images of γ’ morphologies (**b**–**f**) for SL (**a**–**c**) and SH (**d**–**f**) after heat treatment.

**Figure 3:**
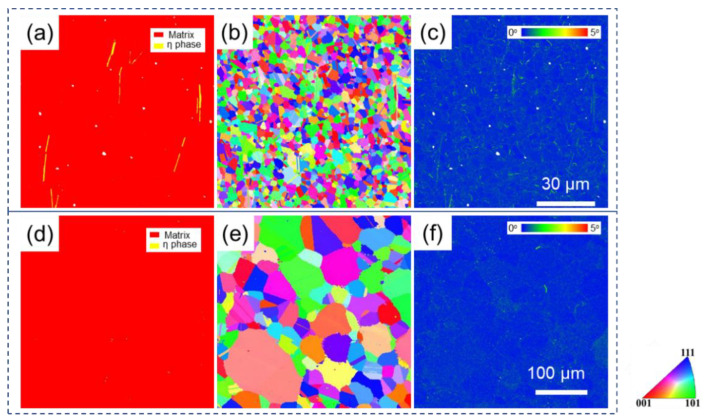
Phase map (**a**,**d**), Inverse pole figure (**b**,**e**), Kernel Average Misorientation map (**c**,**f**), for undeformed SL (**a**–**c**) and SH (**d**–**f**).

The microstructure of the alloy before heat treatment, which was taken by optical microscopy, is shown in Figure 1. It can be observed that the grain size before heat treatment is quite small (~1 μm). The microstructures of the alloy and γ′ phase morphologies after the two heat treatments are shown in Figure 2. It can be seen that the microstructures of the two specimens are quite different after sub-solvus and super-solvus treatments, suggesting the significant effect of solution treatment temperature on microstructure of superalloy. The grain size of the SL subjected to sub-solvus treatment (see Figure 2a) is much smaller compared to that of the SH subjected to super-solvus solution treatment (see Figure 2d). The bright phase is characterized by a long strip, and is about 30 μm in length in SL. However, this phase is not observed in SH. The chemical compositions of the white stripe phase were detected by EDS and are listed in Table 2. It can be seen from Table 2 that the white stripe phase is rich in Ta and Ti compared to the nominal composition of alloy (see Table 1). The white stripe phase can be identified as the η phase according to its chemical composition [38,39], and phase map as detected by EBSD is shown in Figure 3a. In terms of γ′ morphologies for two specimens, primary γ′ phase in SL is an irregular blocky shape (Figure 2b) and is larger compared to that in SH (Figure 2e). The size of the primary γ′ phase in SL is ~3 μm, while it is ~0.8 μm in SH. The primary γ′ phase distributes along the grain boundaries for both alloys. In higher resolution images, the secondary γ′ phase can be observed in both specimens (Figure 2c,f). The secondary γ′ phase is characterized by spherical particles and distributes inner grains for both specimens evenly. The size of the secondary γ′ phase in SL (~0.1 μm) is smaller than that in SH (~0.2 μm), as seen clearly in Figure 2c,f).

The phase map, inverse pole figure (IPF), and kernel average misorientation (KAM) map for alloy after two treatments are shown in Figure 3. The long white stripe-shape phase can be verified to be the η phase in SL by a phase map (Figure 3a), and the η phase was not detected in SH. IPFs for both specimens are shown in Figure 3b,e. No texture can be observed for both specimens, and grain sizes are statistically measured to be 2.72 μm and 35.07 μm for SL and SH, respectively. The KAM values are very low for both specimens after heat treatments, which can be seen clearly in Figure 3c,f.

**Figure 4:**
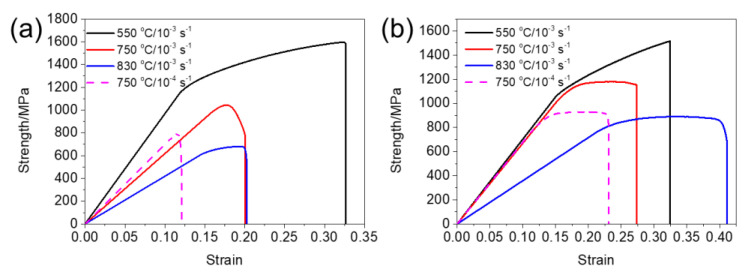
Stress–strain curves for SL (**a**) and SH (**b**) between 550 °C and 830 °C under different strain rates.

Stress–strain curves for two specimens at 550 °C, 750 °C, and 830 °C are shown in Figure 4. It can be seen from Figure 4a that yield strength (YS), ultimate tensile strength, (UTS) and plastic strain to fracture for SL at 550 °C under 10^−3^ s^−1^ strain rate reaches 1168 MPa, 1593 MPa, and 0.16, respectively, exhibiting the highest strength and plastic strain to fracture, among other conditions. With testing temperature increasing, strength and plastic strain to fracture for SL decreased significantly under a strain rate of 10^−3^ s^−1^. Yield strengths for SL at 750 °C and 830 °C under a 10^−3^ s^−1^ strain rate are 1005 MPa and 626 MPa, which decrease 13.96% and 46.40%, respectively, compared to that at 550 °C. Plastic strain to fracture for SL at 750 °C under a 10^−3^ s^−1^ strain rate is measured to be 0.07, which is only 43.75% of that at 550 °C. When the temperature reaches 830 °C, plastic strain to fracture for SL is measured to be 0.05. For evaluating the effect of oxidation on the mechanical performance at a high temperature, the tensile test at 750 °C under a slower strain rate (10^−4^ s^−1^) was also conducted. It can be seen from Figure 4a that a slower strain rate caused destructive damage to the plastic strain to fracture of the alloy, showing barely any plastic strain to fracture at 750 °C. The yield strength also dropped to 790 MPa at 750 °C under a 10^−4^ s^−1^ strain rate.

Similar to SL, a strength drop can also be seen in SH with a temperature increase, as seen clearly in Figure 4b. Yield strengths for SH at 550 °C and 830 °C under a 10^−3^ s^−1^ strain rate are 1089 MPa and 796 MPa, respectively, suggesting a 26.91% drop from 550 °C to 830 °C. The ductility for SH at 550 °C, 750 °C, and 830 °C under a 10^−3^ s^−1^ strain rate are measured to be 0.11, 0.10, 0.17, respectively. In terms of the strain rate, the yield strengths for SH at 750 °C under a 10^−3^ s^−1^ and 10^−4^ s^−1^ strain rate are 1064 MPa and 865 MPa; The plastic strain to fracture at 750 °C under 10^−3^ s^−1^ and 10^−4^ s^−1^ strain rate are 0.10 and 0.09, suggesting a ductility loss with a decrease in the strain rate.

**Figure 5:**
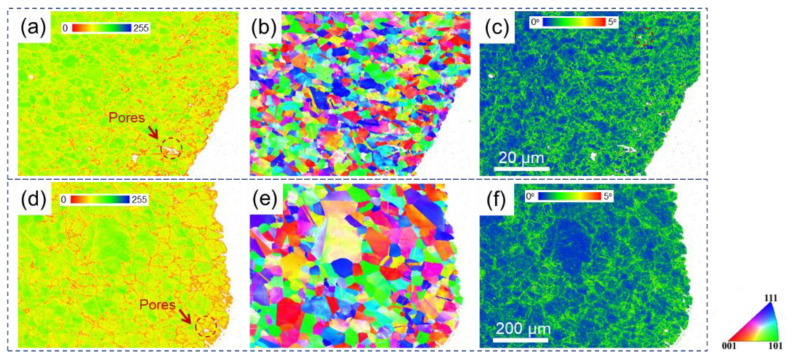
BC (**a**,**d**), IPF (**b**,**e**), and KAM (**c**,**f**) maps of SL (**a**–**c**) and SH (**d**–**f**) near fracture at 550 °C.

Figure 5 shows band contrast, inverse pole figure, and kernel average misorientation maps for two specimens near fracture at 550 °C. It should be noted that the red color in the BC maps represents the lowest quality (BC = 0) of band contrast, while the blue color represents the highest quality (BC = 255) of band contrast. In terms of KAM maps, the blue color represents the lowest local misorientation (KAM = 0°), while the red color represents the highest local misorientation (KAM = 5°). It can be observed from Figure 5a,d that the low band contrast points are mainly concentrated along grain boundaries for both specimens. Note that the inner grains for SH (Figure 5d) show relatively low quality compared to those for SL (Figure 5d), especially near the fracture surface. Besides, some pores can be seen from the BC maps for both specimens near the fracture. The KAM distribution for both specimens after fracture at 550 °C can be seen in Figure 5c,f. It can be observed from Figure 5c,f that the green points, which indicate relatively high local misorientation, are widely spread on the maps for both specimens, indicating severe deformation near the fracture area.

**Figure 6:**
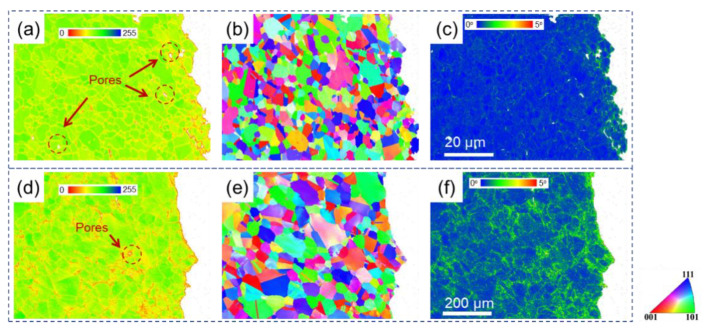
BC (**a**,**d**), IPF (**b**,**e**), and KAM (**c**,**f**) maps for SL (**a**–**c**) and SH (**d**–**f**) near fracture at 750 °C.

BC, IPF, and KAM maps for two specimens near fracture at 750 °C are shown in Figure 6. With the deformation temperature increasing, the microstructures near fracture are quite different from those at 550 °C (Figure 5). The band contrast qualities increased, while KAM values decreased for both specimens, which were tested at 750 °C compared to at 550 °C. However, more pores can be observed near fracture for SL at 750 °C than at 550 °C and the level for SH remained low, which can be seen in Figure 6d. As for the KAM maps for the two specimens at 750 °C, the local misorientation is obviously lower for SL (Figure 6c) than for SH (Figure 6f). The local misorientation for SL is higher at ~5 μm away from fracture and remained at a low level further away from fracture, compared to the undeformed SL (Figure 3c). In contrast, high local misorientations could still be observed at a distance of about 600 μm away from fracture (see Figure 6f).

**Figure 7:**
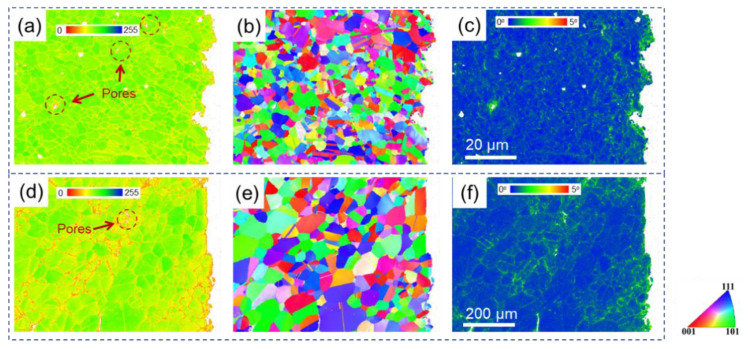
BC (**a**,**d**), IPF (**b**,**e**), and KAM (**c**,**f**) maps of SL (**a**–**c**) and SH (**d**–**f**) near fracture at 830 °C.

Figure 7 shows BC, IPF, and KAM maps for two specimens near fracture at 830 °C. As the testing temperature increased to 830 °C, the BC and KAM maps for SL were quite similar to those at 750 °C, but more pores were generated during the deformation, which can be seen in Figure 7a. As for the KAM map of SH at 830 °C, a lower local misorientation can be observed compared to that at 750 °C (Figure 6f), evenclose to fracture.

**Figure 8:**
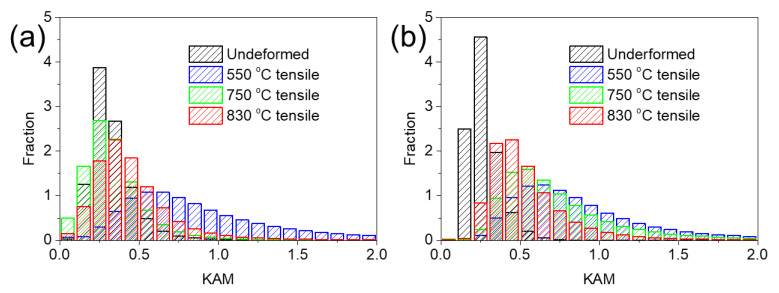
Frequency distribution histograms of the KAM values of SL (**a**) and SH (**b**).

The frequency distribution histograms of the KAM values for both specimens are shown in Figure 8. For SL after tension at 550 °C (Figure 8a), the KAM values are obviously higher than those for an undeformed specimen. On the other hand, the KAM values of the specimen after tension at 750 °C and 830 °C are comparable to those of an undeformed specimen. The KAM distribution difference for SL after deformation at 550 °C, 750 °C, and 830 °C can be seen intuitively from Figure 5, Figure 6 and Figure 7c, in which a higher KAM level is widely distributed at 550 °C but remains low at both 750 °C and 830 °C, compared to the undeformed specimen (Figure 3c). For SH after deformation at all three temperatures, three KAM distributions all drifted to the right from those of the undeformed specimen, which can be seen in Figure 8b. In addition, with an increase in deformation temperature, the KAM distribution histograms moved towards the left side, indicating lower local misorientations after deformation at a higher temperature.

**Figure 9:**
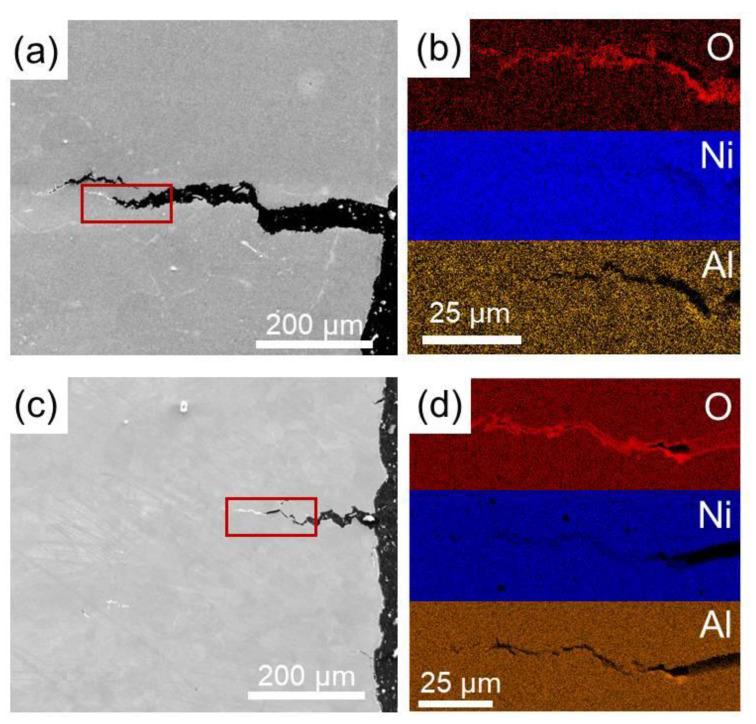
SE images (**a**,**c**) and element distribution maps (**b**,**d**), of the cross-sections for SL (**a**,**b**) and SH (**c**,**d**).

SE images and element distribution maps from the view of a cross-section from both specimens are shown in Figure 9. Cracks generation and propagation can be clearly seen from Figure 9a,c, and the length of cracks in SL were much longer compared to that in SH. In order to evaluate the environmental effect to the deformation behavior at a high temperature, distributions of O, Ni, and Al were detected by EDS around cracks. An enrichment of O along cracks can be seen from Figure 9b,d.

**Figure 10:**
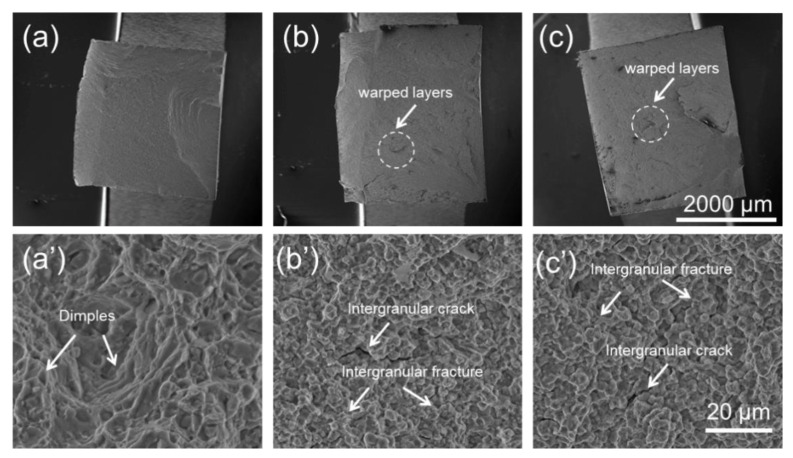
Fracture surfaces for SL at 550 °C (**a**,**a’**), 750 °C (**b**,**b’**), and 830 °C (**c**,**c’**).

The fracture surfaces of SL at 550 °C, 750 °C, and 830 °C are shown in Figure 10. Macro fractographies can be seen from Figure 10a–c, and the corresponding high-resolution images are also given in (a’–c’), respectively. It can be seen from Figure 10a–c that the fracture surface at 550 °C is relatively intact, but some warped layers show up when testing at 750 °C. With an increase in testing temperature to 830 °C, warped layers are more severe compared to those at 750 °C, which can be seen clearly in Figure 10c. With SE images taken with a high resolution (Figure 10a’–c’), more details about the fracture surface can be observed. Dimples are dominant on the fracture surface at 550 °C (Figure 10a’), but can hardly be seen on that at both 750 °C and 830 °C (Figure 10b’,c’). Instead of dimples, intergranular fractures and intergranular cracks can be observed on the fracture surface at 750 °C and 830 °C. The fractographies in the present study are similar to the work of Tan et al. [40], in which the fracture is ductile at a low temperature and brittle at a high temperature.

**Figure 11:**
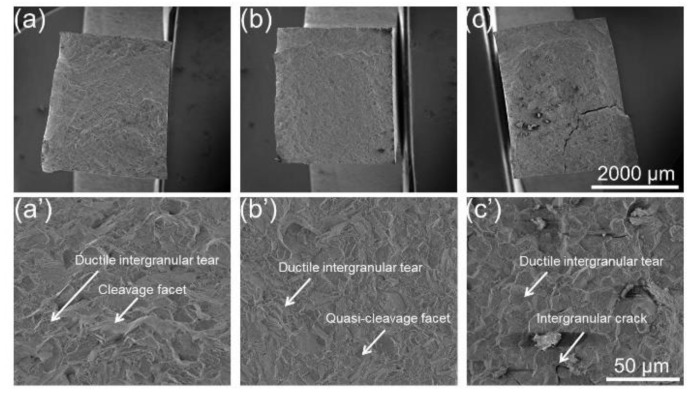
Fracture surfaces of SH at 550 °C (**a**,**a’**), 750 °C (**b**,**b’**), and 830 °C (**c**,**c’**).

The fracture surfaces of SH at 550 °C, 750 °C, and 830 °C are shown in Figure 11. Compared to the fracture surfaces for SL Figure 10a–c, no warped layers can be seen on fracture surfaces of SH Figure 11a–c, which is shown relatively intact. Cleavage facets are obviously seen on the fracture surface at 550 °C, but become less so with an increase in the testing temperature. On the contrary, ductile intergranular tears can be observed for all three samples (Figure 11a’–c’).

**Figure 12:**
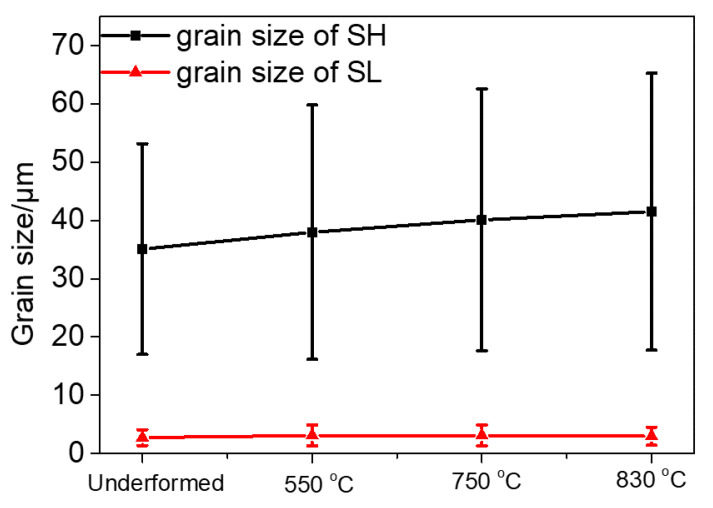
The grain sizes of undeformed and deformed specimens.

The mean grain sizes of the undeformed and deformed samples were statistically measured by EBSD and are presented in Figure 12. The grain size of SH is influenced significantly by deformation and the deformation temperature compared to that of SL. The average grain size of the undeformed SH sample was measured to be 35.07 μm, which was the lowest among the four SH samples. With an increase in the deformation temperature, the grain size became higher and reached 41.51 μm after deformation at 830 °C. Compared to SH, the average grain sizes for SL did not vary greatly through deformation.

## 4. Discussion

From the aforementioned results, the present superalloy with sub-solvus treatment exhibited extremely fine grains and a large primary γ′ compared to the alloy with super-solvus treatment. The specimen with fine grain demonstrated higher strength and higher plastic strain to fracture levels at a low temperature (550 °C) compared to the specimen with large grains, but a significant reduction in both strength and plastic strain to fracture was observed at a higher temperature (750 °C and 830 °C).

### 4.1. Effect of Solution Treatment on Microstructures of Superalloy

During the solution treatment for superalloy, the secondary γ′ phase dissolved into the matrix at a high temperature and precipitated from the matrix during the following cooling process [17]. The super-solvus treatment was carried out above a γ′ solution temperature of alloy, providing a sufficient driving force for γ′ to dissolve into the matrix. On the contrary, the sub-solvus was carried out below the γ′ solution temperature, where only part of the primary γ′ phase dissolved into the matrix [24]. It can be seen from Figure 2b that the primary γ′ phase was dominant along the grain boundary after sub-solvus treatment at 1120 °C for 4 h. A few of the primary γ′ could still be observed after super-solvus treatment at 1180 °C for 4 h (Figure 2e), but exhibited quite small and had little quantity compared to that after sub-solvus treatment. A high treatment temperature for super-solvus accelerated the γ′ dissolution rate and promoted the solubility of γ′ in the matrix, resulting in a relatively uniform γ′ distribution in the matrix, which can be seen clearly in Figure 2e.

It has been widely proven that the secondary phase at grain boundaries could impede grain boundary movements [24,41], hence reducing grain growth. Blocky large primary γ′ at grain boundaries after sub-solvus treatment may be the reason why grains in SL were very small (Figure 3b) after solution treatment compared to SH (Figure 3e). Because of the full dissolution of γ′ during the super-solvus treatment, the grain size in SH could grow without the impediment of γ′, reaching to about 40 μm. 

Besides smaller grain size and larger γ′ in SL compared to SH, a strip-shape η phase could be observed in SL, which could hardly be seen in SH. It has been found that γ′ is thermodynamically unstable at a high temperature and would transform to η phase [42]. Hence, a solid phase transformation from γ′ phase to η phase is expected at 1120 °C. When the treatment temperature increases to 1180 °C which is above the solution temperature, the solid solution γ phase is thermodynamically favorable in alloy, suggesting neither the γ’ nor η phase would form at 1180 °C. In addition, because of the heat treatment before the tensile test, dislocations in alloy have been fully reduced. Hence, the KAM values are very low for both specimens after heat treatments, which can be seen clearly in Figure 3c,f.

### 4.2. Effect of Solution Treatment on Mechanical Performance at High Temperature

It can be seen from Figure 4 that flow stress curves exhibit a steady stage at a high temperature (830 °C) for both specimens, indicating a balance of work hardening and softening. Dynamic recrystallization is not obvious, due to the low temperature of the tensile test [43]. Hence, dynamic recovery may dominate the softening mechanism. It can be seen from Figure 8 that the KAM values remain low for both specimens when deformed at high temperatures (750, 830 °C). The low level of KAM can be ascribed to the significant dynamic recovery at a high temperature, resulting in dislocation annihilation. The strain rate sensitivity can be calculated through:

(1) $$m=\frac{\partial \ln\sigma }{\partial \ln\dot{\varepsilon }}$$

The strain rate sensitivity for SL and SH at 750 °C were calculated to be 0.121 and 0.104, respectively, using the data in Figure 3. It has been reported that the closer strain rate sensitivity isto 0.5 [44], the more pronounced GBS is in the alloy deformation mechanism. The value of the train rate sensitivity for SL is greater than that of SH, indicating a greater part of GBS in SL during deformation compared to SH.

In terms of strength and ductility at a high temperature, SL with small grains showed high strength and ductility at a relatively low temperature. However, with the testing temperature increasing, brittle fracture occurred for SL. It has been well-known that deformation transforms from slipping to grain boundary sliding when deformed at a high temperature [31,45,46,47]. It can be seen from Figure 5a,d that some pores were produced near fracture after deformation at 550 °C. With deformation temperature increasing, pores are more prevalent near fracture, which can be clearly observed in Figure 6 and Figure 7a,d. This may be ascribed to the grain boundary sliding during deformation, resulting in pores at triple points of grain boundaries. It has been reported that alloys with small grains are more inclined to grain boundary sliding compared to alloys with larger grains in the same condition [37,48,49]. It can be verified from the results in the present research, which displays more pores in SL compared to SH. In terms of ductility at a high temperature, SL is more brittle than SH at 750 °C and 830 °C but shows higher ductility at 550 °C. In the case of creep deformation, the deformation is very slow, so that a pore can be refilled by the diffusion of elements, resulting in high ductility [34,46,50]. However, the strain rate (10^−3^ s^−1^) in the present research is much higher compared to the creep deformation, hence pores cannot be filled up by diffusion, resulting in brittle fracture. Hence, it can be inferred that, at low temperature, the Hall–Petch effect dominates the deformation mechanism, resulting in fine grain hardening for SL. However, at a high temperature, grain boundary sliding would dominate the deformation mechanism, resulting in destructive damage to the strength and ductility of SL, owing to its high grain boundary density. In terms of SH, it shows ductile fracture at a high temperature, which can be seen clearly in Figure 11. The plastic strain to fracture level of SH at 750 °C is comparable to that at 550 °C. However, a significant increase in plastic strain to fracture at 830 °C can be observed in Figure 4. This can be ascribed to the strong dynamic recovery in SH at 830 °C, resulting in a low dislocation density in SH. This can be verified by the KAM distributions shown in Figure 8b, in which KAM at 830 °C is the lowest among three test conditions. 

In addition, the grain growth of SH can be observed in Figure 12 during the tensile test at high temperature, and the grain size increased with an increase in the test temperature. The grain growth of SH can be ascribed to the grain boundary migration in a stressed condition at a high temperature [51]. However, the grain growth of SL during the tensile test at high temperature is not obvious, which can be seen in Figure 12. This may be ascribed to the large blocky γ′ along grain boundaries in SL (see Figure 2b), impeding the migration of grain boundaries.

### 4.3. Oxidation Assisted Brittleness for Superalloy

It can be seen from Figure 4 that both SL and SH show less ductility under the strain rate of 10^−4^ s^−1^ at 750 °C compared to that under the strain rate of 10^−3^ s^−1^. This is similar to the results in the study of Németh [52], where it was proved that oxidation would cause deleterious effects on the mechanical performance of alloy at a high temperature. In the present study, oxygen intrusion can be observed in Figure 9b,d, resulting in a high concentration of O along the cracks for both specimens. It can be inferred that oxygen would penetrate to alloy the matrix along the grain boundaries and accelerate the propagation of cracks [53]. Hence, both specimens at 750 °C are more brittle with a slower strain rate due to the longer oxidation time. In addition, ductility loss due to oxidation for SL is more obvious than that for SH, which can be clearly seen from Figure 4. More severe ductility loss for SL may be ascribed to the higher boundary density in SL, resulting in more channels for oxygen intrusion when testing at a high temperature.

**Figure 13:**
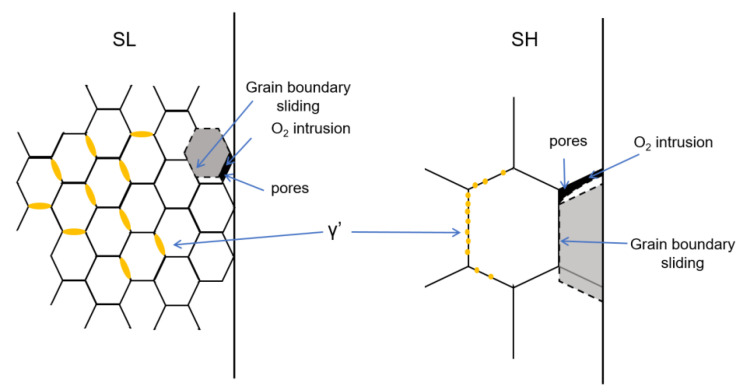
Schematic illustration of heat treatment to mechanical performance of superalloy at high temperature.

Based on the above results and discussion, the schematic illustration of heat treatment to microstructure and the mechanical performance of the novel superalloy at high temperature is drawn in Figure 13. The grains in SL are small due to the impediment of γ′, which is not dissolved into matrix under the solution temperature of γ′. On the contrary, γ′ is fully dissolved in SH after super-solvus treatment, resulting in larger grains in SH compared to those in SL. Because of the transformation of the deformation mechanism from dislocation sliding to grain boundary sliding, more pores at triple points of grain boundaries are generated in SL, owning more grain boundaries at a high temperature compared to SH. Hence, SL shows brittle fracture at a high temperature and lower strength than SH. But SL still owns higher strength and ductility at a low temperature, possibly due to grain refinement hardening. In addition, oxygen intrusion at a high temperature would also accelerate cracks propagation in alloy, resulting in brittle behavior at a high temperature. Because of a high grain boundary density in SL, oxidation assisted brittleness is more obvious in SL.

## 5. Conclusions

Sub-solvus and super-solvus solution treatments were conducted on a novel PM superalloy, suggesting a great influence from solution temperature on the microstructure and corresponding mechanical performance at a high temperature. The following conclusions can be drawn: Sub-solvus treatment produces small grains in superalloy, ascribed to the impediment of large blocky γ′ along grain boundaries, while super-solvus treatment produces about ten times larger grains than that after sub-solvus. Specimens after sub-solvus treatment exhibit higher ductility and strength at 550 °C compared to those after super-solvus. However, a significant drop in strength and ductility can be observed for specimens after sub-solvus at higher temperatures (750 °C, 830 °C). KAM values remain low for both specimens when deformed at high temperatures (750, 830 °C). The low level of KAM can be ascribed to the significant dynamic recovery at a high temperature, resulting in dislocation annihilation. Oxidation at a high temperature leads to strength and ductility loss for both specimens. Note that oxidation damage is more significant for specimens after sub-solvus, which may be ascribed to their high grain boundary density.

## Figures and Tables

**Table 1:** Nominal composition (wt.%) of the novel PM superalloy.

Co	Cr	Mo	W	Al	Ti	Nb	Ta	Hf	B	C	Zr	Ni
18.0	12.0	3.0	3.0	3.0	3.0	1.5	4.5	0.4	0.04	0.04	0.05	Bal.

**Table 2:** Chemical composition of η phase.

	Ni	Co	Ta	Cr	Ti	Al	Mo	Nb
Weight fraction	50.0	15.0	9.9	7.4	4.4	2.7	2.0	1.9
Atomic fraction	55.4	16.56	3.56	9.26	5.98	6.51	1.36	1.33

## Data Availability

Data available on request from the authors.
